# Effects of High-Intensity Interval Training with Blood Flow Restriction Versus Normobaric Hypoxia on Physiological Parameters in Apparently Healthy Young Men

**DOI:** 10.3390/sports14060232

**Published:** 2026-06-05

**Authors:** Jose Jairo Narrea Vargas, Antonio Castillo-Paredes, Alexander Javier Iman Torres, Michelle Lozada-Urbano, Delsi M. Huaita Acha, Felipe Montalva-Valenzuela, Gustavo Humeres, Diego A. Bonilla

**Affiliations:** 1 Grupo de Investigación en Nutrición, Metabolismo y Ejercicio, Facultad de Ciencias de la Salud, Carrera de Nutrición y Dietética, Universidad Científica del Sur, Lima 15067, Peru; 2 Grupo AFySE, Investigación en Actividad Física y Salud Escolar, Escuela de Pedagogía en Educación Física, Facultad de Educación, Universidad de Las Américas, Santiago 8370040, Chile; acastillop85@gmail.com; 3 Departamento de Ciencia y Tecnología de Alimentos, Facultad de Industrias Alimentarias, Universidad Nacional de la Amazonía Peruana, Iquitos 16001, Peru; alexander.iman@unapiquitos.edu.pe; 4 Programa Académico en Nutrición y Dietética, Universidad Privada Norbert Wiener, Lima 15046, Peru; michelle.lozada@uwiener.edu.pe; 5 Dirección General de Investigaciones, Universidad Inca Garcilaso de la Vega, Lima 15084, Peru; delsi.huaita@uigv.edu.pe; 6 Escuela de Entrenador en Actividad Física y Deporte, Facultad de Ciencias Humanas, Universidad Bernardo O’Higgins, Santiago 8370040, Chile; felipe.montalva@ubo.cl; 7 Research Group in Elite Sports Performance (DBSS-HiPer), Dynamical Business & Science Society—DBSS GLOBAL SAS, Buenos Aires C1032AAM, Argentina; ghumeres@dbss.pro; 8 Research Group in Integrative Physiology (DBSS-Phi), Dynamical Business & Science Society—DBSS International SAS, Bogota 110311, Colombia; dabonilla@dbss.pro

**Keywords:** exercise physiology, metabolism, stress, physiological

## Abstract

High-intensity interval training (HIIT) is an efficient exercise strategy capable of eliciting acute cardiovascular and metabolic responses. Blood flow restriction (BFR) and normobaric hypoxia (NH) have been proposed as exposures to intensify physiological stress during exercise; however, comparative evidence between both strategies remains limited. The aim of this study was to compare acute physiological responses in HIIT protocols performed under BFR and NH in apparently healthy young men. Eight volunteers completed two HIIT sessions in this fixed-order within-subject repeated-measures study: one with BFR and one with NH (simulated altitude: 3536 m above sea level; FiO_2_ ≈ 13.5%). Blood glucose, peripheral oxygen saturation (SpO_2_), heart rate, and blood pressure were repeatedly measured during the exercise protocol. After Bayesian analysis, no evidence of differences in glycemic response was observed, while heart rate and diastolic blood pressure responses appeared broadly comparable between conditions. However, SpO_2_ was consistently lower during NH, whereas systolic blood pressure values were higher under BFR. Although both BFR and NH induced acute physiological responses characteristic of HIIT, distinct physiological profiles were observed. NH was associated with greater systemic hypoxemic stress, whereas BFR showed higher systolic pressor responses.

## 1. Introduction

High-intensity interval training (HIIT) has emerged as a time-efficient exercise modality capable of inducing cardiorespiratory and metabolic adaptations comparable to moderate-intensity continuous training, with lower time demands [1]. The capacity to augment physiological stress during HIIT without proportionally increasing mechanical load has become a relevant question in exercise prescription, motivating interest in strategies such as blood flow restriction (BFR) [2] and normobaric hypoxia (NH) [3].

BFR involves the application of external pressure to the limbs to partially restrict venous return and, to a lesser extent, arterial perfusion during exercise. When combined with continuous and interval training, BFR improves cardiorespiratory fitness even at lower exercise intensities [4]. When applied specifically during HIIT, BFR has been shown to improve maximal oxygen uptake, maximal aerobic power, and exercise tolerance relative to normoxic HIIT without restriction [5,6]. These effects are attributed to the hemodynamic and metabolic consequences of localized muscular hypoxia induced by vascular occlusion [7,8,9]. Clinical trials have suggested that the application of BFR during HIIT may modulate peripheral fatigue, increase muscle fiber recruitment, and generate hemodynamic stimuli without requiring greater global mechanical load [8,10]. These responses have been proposed as potential mechanisms contributing to vascular and endothelial adaptations, although such mechanisms were not directly assessed in the present study [11].

Interval training under NH conditions, in contrast, reduces systemic oxygen availability by lowering the fraction of inspired oxygen without altering barometric pressure. Exercise under hypoxic conditions has been proposed to induce cardiorespiratory and metabolic adaptations through greater reliance on glycolytic pathways and activation of hypoxia-sensitive signaling responses, potentially including angiogenic-related mechanisms [12]. The magnitude of these adaptations depends on the degree of hypoxia, exposure duration, and the training status of the individual [12,13].

Despite their shared capacity to limit oxygen availability during exercise, BFR and NH differ in the site and mechanism of oxygen restriction: BFR induces localized peripheral hypoxia through vascular occlusion, whereas NH reduces systemic arterial oxygen content through a reduction in inspired oxygen fraction. Evidence agrees that both exposures generate lower oxygen availability at the muscular level during HIIT, although with important differences in cardiovascular and metabolic capacity [8,14]. Previous investigations comparing localized flow restriction and systemic hypoxia during repeated-sprint exercise have shown partially overlapping physiological responses but important differences in oxygenation and cardiovascular regulation [14,15], supporting the need for further direct comparisons under standardized HIIT protocols.

Although both interventions reduce oxygen availability during exercise, the physiological stress induced by each strategy differs substantially. BFR primarily produces localized muscular hypoxia through partial vascular occlusion, increasing metabolic stress within the active musculature while preserving systemic arterial oxygenation [4,7]. In contrast, NH induces systemic hypoxia by reducing inspired oxygen fraction, leading to lower arterial oxygen saturation and greater whole-body oxygen stress [12]. Consequently, the adaptive responses associated with each intervention may arise from different physiological pathways despite partially overlapping exercise responses.

To our knowledge, few studies have directly compared both strategies during an acute, standardized HIIT session in individuals unacclimatized to altitude, limiting the ability to determine which modality imposes greater systemic or local physiological stress in this population. Therefore, the aim of the present study was to compare the acute physiological responses to a single HIIT session under BFR and NH in apparently healthy young men residing below 1000 m above sea level. We hypothesized that NH would elicit greater systemic desaturation than BFR (lower SpO_2_), whereas BFR would impose a higher hemodynamic load (higher SBP), with comparable responses between conditions in HR, DBP, and capillary glucose.

## 2. Materials and Methods

### 2.1. Study Design

This study employed a fixed-order within-subject repeated-measures design to compare the acute effects of a single HIIT session under NH and BFR conditions. The order of conditions was fixed, with all participants completing NH first, followed by BFR, separated by a four-day washout period to reduce the carryover effect [16]. This non-randomized sequence was selected for logistical and safety considerations related to hypoxic exposure control; however, a potential order effect cannot be completely excluded. This design was selected to control for interindividual variability and to enhance statistical power with a reduced sample size. The intervention procedures and reporting structure for this fixed-order within-subject repeated-measures study were described following the Template for Intervention Description and Replication (TIDieR) recommendations to improve transparency and reproducibility of the experimental protocol [17].

Due to the nature of the interventions, blinding of participants and assessors was not feasible. Participants were aware of the intervention condition because NH and BFR involve distinct perceptual and procedural characteristics, and outcome assessments were conducted by the research staff supervising the exercise sessions.

### 2.2. Setting

The study was conducted at the Altitude Tolerance Laboratory of the Universidad Peruana Cayetano Heredia (Lima, Peru). Participants attended four sessions: two for determination of maximal power (W_max_) output (phase 1) and two for completion of the HIIT protocols under each condition (phase 2). The time between the W_max_ tests and the HIIT sessions was one week. The HIIT sessions were separated by a four-day washout period [18]. The study was approved by the Ethics Committee of the Universidad Privada Norbert Wiener (code: 059-2020) and conducted in accordance with the principles of the Declaration of Helsinki [19]. The characteristics, purpose, risks, and confidentiality of the study were explained to each participant both verbally and in writing, after which written informed consent was obtained prior to enrollment.

### 2.3. Participants

University students residing in Lima (Peru) responded to the call to participate in this study. Inclusion criteria included the following: age 18–30 years; body mass index (BMI) < 30 kg/m^2^ and body fat ≤ 24% assessed by bioelectrical impedance analysis [20]; residence below 1000 m above sea level; low-to-moderate physical activity level according to the IPAQ-SF; and low cardiovascular risk based on the PAR-Q+ [21]. Exclusion criteria were the following: smoking; pulmonary, renal, endocrine, musculoskeletal, cardiovascular, or neurological disorders; regular participation in competitive sports or ≥12 weeks of prior interval training [22]; and exposure to altitudes >2000 m within the two months preceding the study [18].

### 2.4. Experimental Procedures

All procedures were supervised by a cardiologist specialized in exercise testing and by trained research staff. Participants were monitored for 45 min following each test and HIIT session to ensure safety. The interval between W_max_ testing and HIIT sessions was seven days for both conditions. HIIT sessions followed the same fixed order (NH then BFR), separated by a four-day washout period [16]. Participants were instructed to refrain from moderate-to-high intensity exercise for 48 h before each visit, to avoid caffeine and alcohol for 24 h, and to arrive after a minimum 4-h fast. Their habitual diet was maintained during the week preceding each session. To standardize hydration, participants consumed approximately 30 mL·kg^−1^·day^−1^ of water for three days prior to testing and 5–10 mL·kg^−1^ two hours before each session [23].

Participants remained seated for five minutes before each session to allow physiological stabilization under each condition. This was followed by a 3-min warm-up, five high-intensity intervals of 1.5 min each, interspersed with 3-min active recovery periods, and a final 3-min recovery (total duration: 22.5 min). High-intensity intervals were performed at 90% of condition-specific W_max_, and recovery periods were performed at 50% of W_max_. Warm-up and final recovery were performed at a fixed load of 0.5 kp [24].

In the BFR condition, a Scientific Leg vascular restriction system (Cefise Biotecnologia Esportiva, Brazil) was applied at the inguinal level using pneumatic cuffs (inflatable component: 7 × 52 cm; cuff sleeve: 12.5 × 84 cm). Arterial occlusion pressure (AOP) was estimated at rest using Doppler ultrasound, and exercise was performed at 80% AOP with an additional 10 mmHg to compensate for potential pressure loss [25]. The mean pressure corresponding to 80% AOP was 190.0 ± 33.6 mmHg (range: 128–224 mmHg), while the final applied pressure averaged 237.5 ± 42.0 mmHg (range: 160–280 mmHg). Pressure was maintained continuously throughout exercise and recovery periods, with continuous monitoring during the intervention. Perceptual responses, including discomfort and rating of perceived exertion, were not systematically recorded. The selected pressure was based on previous exercise protocols employing high-pressure BFR and was continuously monitored throughout the intervention, including recovery intervals to ensure procedural consistency and participant safety [4,25]. In the NH condition, tests were performed using the Hypoxico Everest Summit II normobaric hypoxia generator (Hypoxico Inc., New York, NY, USA), configured to simulate an altitude of 3536 m above sea level, corresponding to an inspired oxygen fraction (FiO_2_) of approximately 13.5%, while maintaining normobaric pressure conditions [26].

### 2.5. Variables

**Figure 1:**
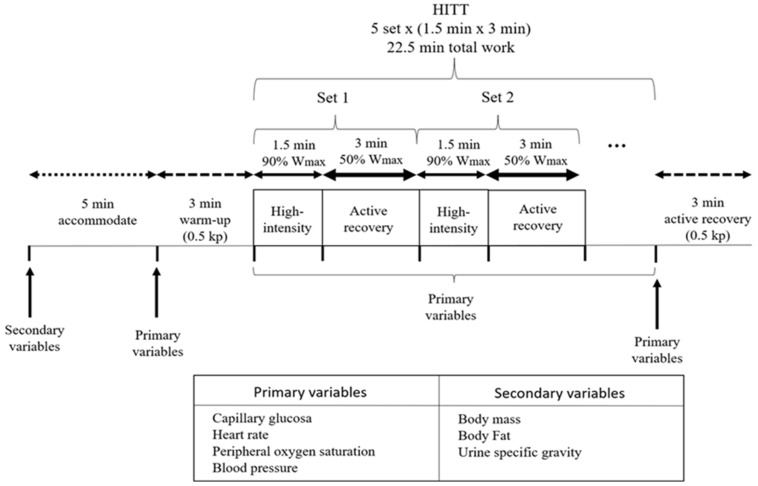
Organization of the HIIT protocol.

The primary outcomes were capillary glucose, heart rate (HR), peripheral oxygen saturation (SpO_2_), systolic blood pressure (SBP), and diastolic blood pressure (DBP). All variables were recorded at the end of each high-intensity interval. HR, SpO_2_, and blood pressure were assessed using the devices described above. Body mass and urine specific gravity were recorded prior to each session as pre-session control variables to verify hydration status and confirm standardization of the protocol conditions. The data collection timeline is presented in Figure 1.

### 2.6. Outcomes

All assessments were performed in the morning (08:00–12:00 h) under controlled environmental conditions (18–20 °C; 50–70% relative humidity). In phase 1, W_max_ was determined through maximal incremental tests performed under each condition (NH and BFR). In phase 2, condition-specific W_max_ values were used to prescribe individualized workloads for the corresponding HIIT sessions.

#### 2.6.1. Anthropometry and Body Composition

Body mass was measured using a Tanita Ironman BC-558 bioelectrical impedance scale (Tanita Corporation, Tokyo, Japan), with a precision of 0.1 kg, while participants were barefoot and wearing light clothing. Stature was measured using a SECA 206 portable stadiometer (SECA GmbH & Co. KG, Hamburg, Germany), with a precision of 0.1 cm. Both measurements were performed following the recommendations of the International Society for the Advancement of Kinanthropometry (ISAK) [27].

Body composition was assessed using the same bioelectrical impedance analyzer (Tanita Ironman BC-558, Tokyo, Japan), following a standardized protocol under controlled conditions, with the addition of prior bladder voiding [28]. Assessments were conducted during morning hours in a controlled environmental setting, in accordance with the manufacturer’s recommendations and standardized international protocols [29]. Urine specific gravity was measured using a refractometer (Uricon-NE, ATAGO Co., Ltd., Tokyo, Japan).

#### 2.6.2. Physical Performance Assessment

W_max_ was determined through a maximal incremental test on a Monark Ergomedic 828E cycle ergometer (Monark Exercise AB, Vansbro, Sweden), calibrated according to the manufacturer’s instructions. Seat height was individually adjusted for each participant to allow knee flexion of 5–10° at the lowest point of the pedaling cycle [30].

The protocol began with a three-minute warm-up at 30 W, followed by increments of 30 W every three minutes. Participants maintained a constant cadence of 60 ± 2 rpm, controlled using a digital metronome (KORG KDM-1, Korg Inc., Tokyo, Japan) and continuous verbal encouragement from the investigator. The test was terminated upon reaching volitional exhaustion or the clinical criteria for test termination established by the American College of Sports Medicine [31]. W_max_ was defined as the highest power output sustained for at least one minute or, if the last stage was not completed, as the power output corresponding to the immediately preceding stage.

#### 2.6.3. Physiological and Metabolic Variables

Continuous monitoring was performed throughout the test and included electrocardiography via the integrated ECG module of the COSMED Quark C12x gas analyzer (COSMED Srl, Rome, Italy), pulse oximetry (Nellcor N-560, Nellcor Puritan Bennett Inc., USA), and intermittent blood pressure measurement using an aneroid sphygmomanometer (Riester Minimus II, Rudolf Riester GmbH, Jungingen, Germany) and a stethoscope (Riester Duplex 2.0, Rudolf Riester GmbH, Jungingen, Germany). Blood pressure was recorded every three minutes in the arm contralateral to the SpO_2_ measurement site. Capillary glucose was measured via fingertip sampling (Accu-Chek Active, Roche Diagnostics).

### 2.7. Sample Size

Non-probability sampling (convenience sampling) was implemented, considering the accessibility of the study population and the inherent difficulty in recruiting volunteers for high-intensity physical exercise protocols [1]. Participants were recruited through written and verbal announcements at a university in Lima, Peru. Of the 31 university students initially contacted, 16 agreed to participate and were potentially eligible.

### 2.8. Statistical Analysis

Baseline characteristics are presented as mean (standard deviation). Additionally, 95% confidence intervals (95% CI) were calculated using the standard formula for small samples, mean ± (t critical value × standard error), with degrees of freedom equal to n − 1, to estimate the precision of the group means. Physiological variables recorded during the HIIT protocol were analyzed using Bayesian repeated-measures ANOVA, with time, condition (BFR vs. NH), and their interaction as within-subject factors, consistent with the within-subject repeated-measures design. Participant was included as a random factor with random slopes specified for repeated-measures factors. For each physiological variable, five competing models were evaluated: null (including participant as a random effect), time, condition, time + condition, and time × condition. Prior model probabilities were set equally across models. Model comparison was based on Bayes factors (BF_10_) and posterior model probabilities (P(M|data)). Evidence was interpreted according to previously established thresholds [32].

To evaluate the contribution of individual predictors, Bayesian inclusion factors (BF incl) and posterior inclusion probabilities (P(incl|data)) were additionally computed. BF incl values were interpreted using the same thresholds applied to BF_10_. All Bayesian analyses were conducted in JASP using default priors to support reproducibility [32]. Bayesian post hoc comparisons, adjusted for multiplicity, were performed when at least moderate evidence was observed (BF_10_ > 3). Data visualization was performed within the R statistical computing environment (v4.5.1) [33]. Raincloud plots were generated using the ggplot2 (v4.0.1) and ggdist (v3.3.3) packages [34,35].

## 3. Results

**Figure 2:**
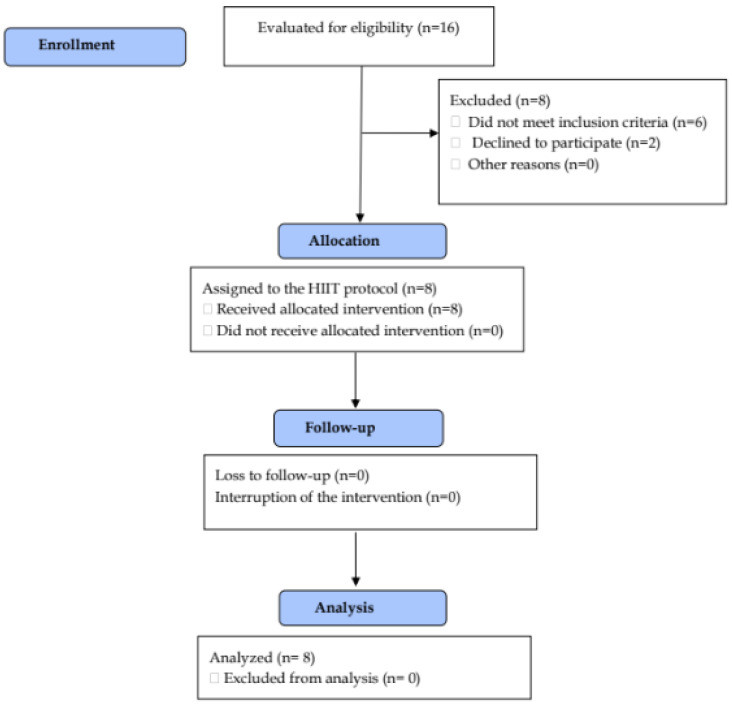
Flow diagram of participant enrollment, allocation, follow-up, and analysis.

A total of 16 participants were assessed for eligibility, of whom eight were excluded: six did not meet the inclusion criteria, and two declined to participate. The remaining eight apparently healthy young men were enrolled and completed both experimental conditions (NH and BFR), with no loss to follow-up or protocol deviations. All participants were included in the statistical analyses. No adverse events were reported during any experimental session. The participant flow diagram is presented in Figure 2.

### 3.1. Baseline Data

Baseline characteristics of the eight participants (24.3 [3.9] years; 75.2 [5.9] kg; 171.3 [7.2] cm; 25.7 [1.7] kg/m^2^), assessed prior to the first experimental session, are presented in Table 1 as means (standard deviation) with 95% CI. Pre-protocol urine specific gravity was within the euhydration range established for the study.

### 3.2. Physiological Responses During the HIIT Protocol

For each physiological variable, five Bayesian repeated-measures ANOVA models were compared, as described in Section 2.8. Model comparison results are presented in Table 2, and inclusion Bayes factors for individual predictors are presented in Table 3.

**Figure 3:**
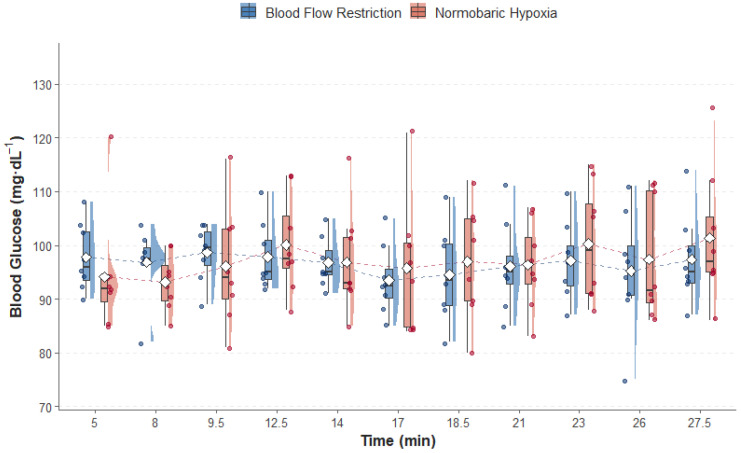
Effect on blood glucose according to the HIIT protocol during BFR and NH exposures in participants. Data are presented as raincloud plots including individual data points, box plots, and density distributions. Dashed lines indicate the group response trajectory connecting repeated measures within the same condition.

For capillary glucose (Figure 3), the null model showed the highest posterior probability (P(M|data) = 0.728). Bayesian inclusion factors favored the exclusion of time, condition, and their interaction, indicating no relevant contribution of these predictors to glycemic response during the HIIT session. Capillary glucose values remained comparable between NH and BFR throughout the protocol.

**Figure 4:**
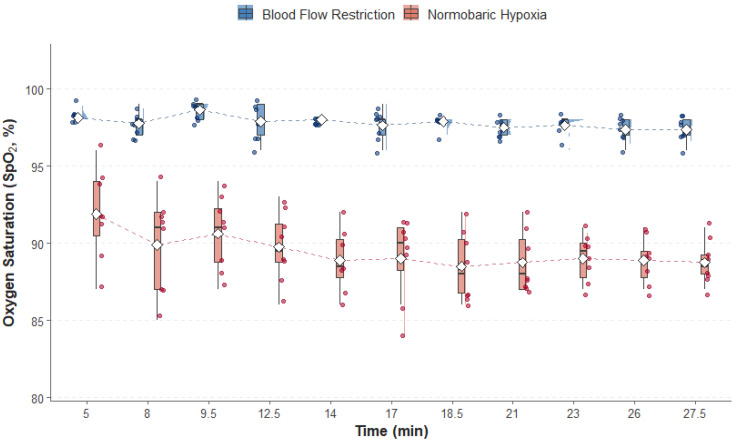
Effect on peripheral oxygen saturation (SpO_2_) according to the HIIT protocol during BFR and NH exposures in participants. Data are presented as raincloud plots including individual data points, box plots, and density distributions. Dashed lines indicate the group response trajectory connecting repeated measures within the same condition.

For SpO_2_ (Figure 4), the time + condition model showed the highest posterior probability (P(M|data) = 0.727). Bayesian inclusion factors supported the effects of condition and time, whereas evidence for the interaction remained limited. SpO_2_ values were consistently lower under NH compared with BFR throughout the protocol, suggesting greater systemic hypoxemia during NH exposure.

**Figure 5:**
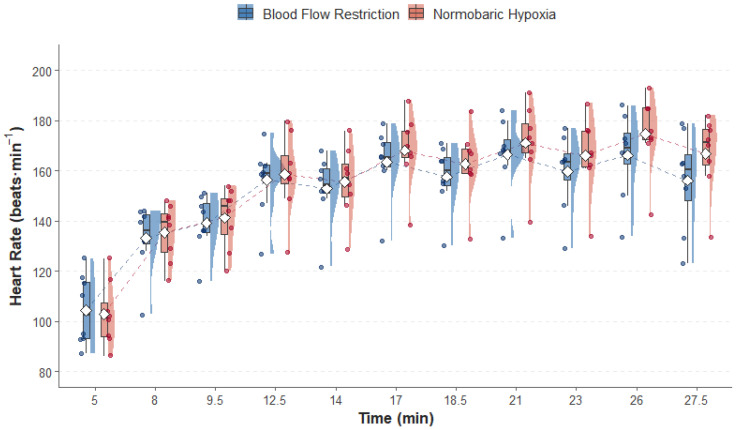
Effect on heart rate according to the HIIT protocol during BFR and NH exposures in participants. Data are presented as raincloud plots including individual data points, box plots, and density distributions. Dashed lines indicate the group response trajectory connecting repeated measures within the same condition.

For HR (Figure 5), the time + condition model showed the highest posterior probability (P(M|data) = 0.878). HR increased progressively across the protocol under both conditions, with evidence supporting independent effects of time and condition but limited evidence for their interaction. Overall, HR responses appeared broadly comparable between exposures.

**Figure 6:**
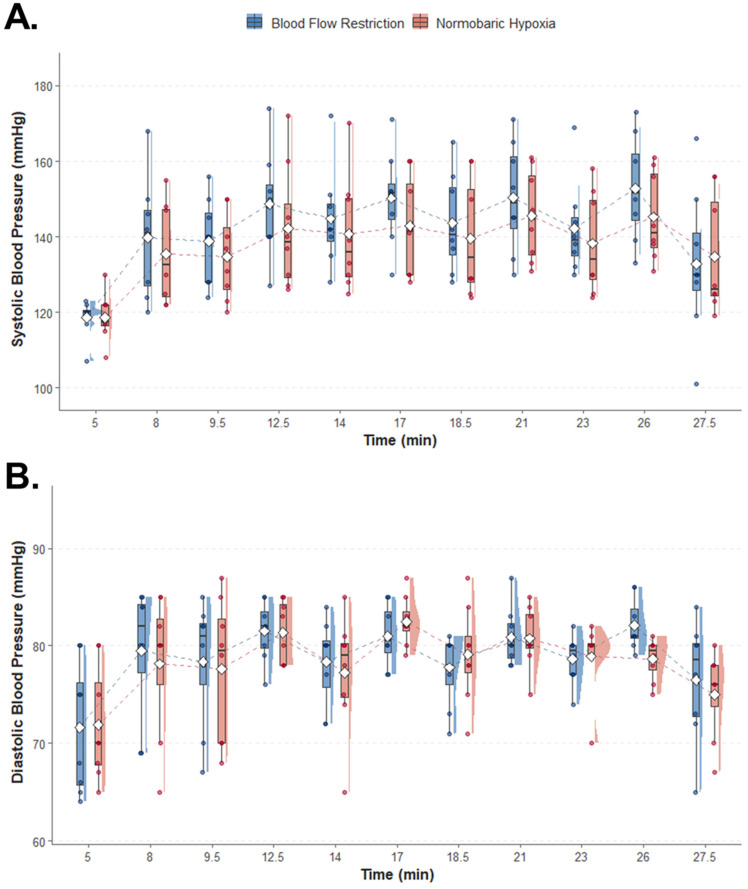
Effect on blood pressure according to the HIIT protocol during BFR and NH exposures in participants. Data for SBP (**A**) and DBP (**B**) are presented as raincloud plots including individual data points, box plots, and density distributions. Dashed lines indicate the group response trajectory connecting repeated measures within the same condition.

For SBP, the time + condition model showed the highest posterior probability (P(M|data) = 0.922), while for DBP the time-only model showed the highest posterior probability (P(M|data) = 0.815). SBP values were consistently higher under BFR than NH throughout the protocol, whereas DBP responses appeared comparable between conditions (Figure 6). Evidence supported independent contributions of time and condition for SBP, while DBP variation was primarily associated with time.

## 4. Discussion

The present study compared, using a within-subject repeated-measures design, the acute physiological responses to a single HIIT session performed under BFR and NH conditions in apparently healthy young men residing below 1000 m above sea level. To the best of our knowledge, few studies have directly compared both hypoxic strategies under a standardized HIIT protocol.

NH induced marked systemic hypoxemia, reflected in consistently lower SpO_2_ values, while BFR elicited higher SBP throughout the protocol. HR and SBP both showed additive contributions of time and exposure type, with no interaction between them: HR was driven primarily by temporal progression, with exposure type contributing a secondary effect, while SBP showed comparable evidential support for both factors independently. DBP was determined exclusively by exercise progression, regardless of exposure type. Capillary glucose remained stable across exposure types and time points, indicating comparable acute glycemic regulation between both hypoxic modalities. Collectively, these findings suggest potentially different physiological responses between systemic hypoxia and local flow restriction during acute HIIT; however, these observations should be interpreted cautiously given the exploratory nature of the study.

Capillary glucose was the only variable for which the null model showed the highest posterior probability, with evidence favoring the exclusion of time, condition, and their interaction. This finding confirms the pre-specified hypothesis of comparable glycemic responses between BFR and NH and indicates that neither the hypoxic modality nor the temporal progression of exercise constituted relevant predictors of glycemic regulation during the acute HIIT session. This pattern of evidence distinguishes capillary glucose from all other variables assessed, in which time or condition were supported as meaningful predictors.

This finding is consistent with prior reports indicating that glycemic regulation during HIIT is governed primarily by relative exercise intensity and active muscle mass, rather than by the nature of the hypoxic stimulus [36,37]. Studies examining glycemic responses under normobaric hypoxia have reported glucose concentrations comparable to normoxic conditions during acute sessions of matched intensity and duration [38], suggesting that reduced systemic oxygen availability does not substantially alter acute glucoregulation during high-intensity exercise. Studies combining BFR with continuous cardiovascular exercise have similarly reported stable glycemic responses despite increased local metabolic stress, a pattern consistent with systemic compensatory mechanisms including enhanced catecholamine release, increased hepatic glucose output, and relative attenuation of peripheral glucose uptake secondary to sympathetically mediated vasoconstriction proximal to the restriction site [39]. The glycemic response during acute HIIT appears to be determined primarily by exercise intensity and duration, with the type of hypoxic exposure contributing negligible additional variance. From a practical standpoint, both BFR and NH produced equivalent acute glycemic profiles in this sample, a finding that may inform exercise prescription in metabolically healthy individuals. Whether this stability is preserved in individuals with impaired glucose metabolism or insulin resistance remains to be established, given that the present sample was restricted to apparently healthy young men and the study was not designed to detect glycemic differences in clinical populations.

The SpO_2_ response showed the clearest separation between conditions throughout the HIIT protocol, with extreme Bayesian evidence supporting the effect of condition. NH induced marked systemic hypoxemia, whereas BFR maintained SpO_2_ within normal physiological ranges, in accordance with the hypothesis stated a priori and consistent with the mechanistic differences underlying each strategy. NH reduces the inspired oxygen fraction (FiO_2_) without altering barometric pressure, thereby decreasing alveolar oxygen partial pressure and arterial oxyhemoglobin saturation at the systemic level [15]. BFR, in contrast, induces localized intramuscular hypoxia through partial vascular occlusion, limiting oxygen delivery to active musculature without altering systemic arterial oxyhemoglobin saturation [4]. The absence of a time × condition interaction, for which only anecdotal evidence for inclusion was observed, indicates that although SpO_2_ levels differed markedly between conditions, the temporal pattern of change was similar under both, suggesting that the systemic hypoxic load imposed by NH remained stable throughout the protocol rather than accumulating progressively.

Previous studies examining BFR during high-intensity exercise under normoxic conditions have reported SpO_2_ values within normal physiological ranges [6], in line with the present observations. Studies conducted under NH at comparable simulated altitudes have reported characteristic reductions in SpO_2_ during exercise [40,41], further supporting the present findings. These results suggest that BFR and NH may induce different systemic oxygenation responses during acute HIIT, a distinction that reflects the divergent sites of oxygen limitation inherent to each strategy and that has direct relevance for their respective physiological and clinical applications.

On the other hand, HR increased progressively throughout the protocol under both experimental conditions, consistent with our hypothesis of comparable HR responses between BFR and NH. Time emerged as the primary predictor of HR variation, supported by extreme Bayesian evidence, with condition contributing an independent but secondary effect. No time × condition interaction was observed, indicating that the temporal trajectory of HR was parallel across conditions. Under NH, this progressive increase may be related to compensatory cardiovascular responses associated with reduced arterial oxygen availability [42]. Although both conditions produced comparable HR responses, previous studies have proposed that the physiological pathways involved may differ between systemic hypoxia and localized vascular restriction [43]. However, autonomic mechanisms were not directly assessed in the present study.

The effects on blood pressure showed different responses. SBP was independently influenced by both the temporal progression of the protocol and condition, with extreme Bayesian evidence supporting the effect of time and strong evidence supporting the effect of condition, consistent with the pre-specified hypothesis that BFR would impose a higher hemodynamic load than NH. SBP values were consistently higher under BFR than NH throughout the protocol, and the absence of a time × condition interaction (evidence favored exclusion) indicates that this differential pressor response was sustained across all measurement timepoints regardless of exercise progression. The elevated SBP observed under BFR may be associated with hemodynamic responses related to external compression and increased peripheral vascular resistance during exercise [4]. Under NH, previously proposed vascular responses associated with systemic hypoxia may contribute to the lower SBP observed in the present study [42,43]. However, vascular function and related physiological mechanisms were not directly assessed.

In contrast, DBP was governed exclusively by the temporal progression of the protocol, with extreme Bayesian evidence supporting the effect of time, while evidence favored the exclusion of both condition effects and interaction terms. This pattern may reflect the typical hemodynamic response observed during HIIT, in which exercise progression is accompanied by cardiovascular adjustments that may contribute to maintaining DBP within comparable ranges across conditions [42]. Regardless of whether hypoxia was systemic (NH) or local (BFR), the vasodilatory mechanisms activated during high-intensity exercise appear to override any exposure-specific effect on DBP, a finding consistent with prior reports [15].

The stability of DBP across both conditions, despite divergent SBP responses, indicates that the hemodynamic profile during acute HIIT under either modality supports the maintenance of adequate diastolic perfusion pressure. Whether this pattern extends to populations with hypertension or elevated cardiovascular risk warrants direct investigation, as coronary perfusion pressure was not assessed in the present study and the sample was restricted to apparently healthy young men.

Overall, the contrasting physiological responses observed between BFR and NH may reflect differences in the site of oxygen restriction. NH reduces systemic oxygen availability, whereas BFR primarily affects local muscular oxygenation through partial vascular occlusion [12,15,43]. These differences may contribute to the distinct oxygenation and blood pressure responses observed despite partially overlapping exercise responses, consistent with previous comparisons of localized and systemic hypoxic stimuli showing differences in the magnitude and site of oxygen restriction [15,44].

### 4.1. Practical Implications

In practical terms, the present findings suggest that HIIT with BFR may induce physiological responses partially comparable to those observed under NH, while showing lower systemic oxygen desaturation. However, these observations were obtained in apparently healthy young men and should be interpreted cautiously. Therefore, extrapolation to therapeutic settings, rehabilitation contexts, or populations with reduced exercise tolerance requires further investigation. Conversely, HIIT under NH may represent a strategy to increase systemic cardiorespiratory stress during exercise, which could be of interest in sports settings, although its application should remain supervised and individualized.

### 4.2. Limitations, Strengths and Future Directions

The small sample size (*n* = 8) and the exclusive inclusion of young, apparently healthy men are considered limitations, which limits the extrapolation of the findings to other populations. In addition, the intervention sequence was fixed rather than randomized, as all participants completed NH before BFR. Although a washout period was implemented, potential order or residual carryover effects cannot be completely ruled out and should be considered when interpreting the findings. Furthermore, neither participants nor assessors were blinded to the intervention condition due to the inherent characteristics of NH and BFR exposures, which may have introduced performance or measurement bias. However, Bayesian inference was selected due to its suitability for within-subject designs and its ability to continuously quantify evidence beyond dichotomous significance thresholds. Nevertheless, the small sample size relative to the complexity of the repeated-measures models requires cautious interpretation, and the findings should be considered exploratory. In addition, the analysis only considered acute physiological effects, without including endocrine, neuromuscular, or perceived effort variables that would help provide a more comprehensive characterization of the stress induced by each exposure. Furthermore, perceptual and tolerability-related responses, including discomfort, rating of perceived exertion, dyspnea, pain perception, and session tolerance, were not systematically recorded. This limits further characterization of the practical tolerability and safety profile of both BFR and NH exposures.

Among the strengths of the study are the within-subject repeated-measures design, which reduced interindividual variability, and the Bayesian analysis, which is appropriate for small samples and for evaluating evidence in favor of both the null and alternative hypotheses. Likewise, the direct comparison between exposures (BFR vs. NH) under a standardized HIIT protocol represents an important academic contribution.

Future studies should consider the chronic evaluation of both exposures using longitudinal and randomized designs, explore different levels of hypoxic severity in NH and restriction pressures in BFR, as well as the location of vascular restriction, and incorporate a broader range of physiological outcomes, including endocrine, respiratory, neuromuscular, and molecular variables. In addition, perceptual and safety-related outcomes, such as rating of perceived exertion, dyspnea, discomfort, pain perception, and session tolerability, should be included to improve the characterization of the practical applicability and tolerability of localized and systemic hypoxic exposures. Where feasible, strategies to reduce observer-related bias should also be considered.

## 5. Conclusions

Acute HIIT performed under BFR and NH elicited partially overlapping physiological responses in apparently healthy young men, with exercise progression contributing substantially to the observed changes. However, distinct responses were observed between conditions, particularly regarding oxygenation and hemodynamic variables, with lower SpO_2_ during NH and higher SBP under BFR. These findings suggest different physiological profiles between both strategies during acute exercise; however, they should be interpreted cautiously given the exploratory nature of the study, the small sample size, and the non-randomized within-subject repeated-measures design. Furthermore, the findings are limited to acute responses in apparently healthy young men and should not be extrapolated to chronic adaptations, clinical populations, or therapeutic settings.

## Figures and Tables

**Table 1:** Baseline characteristics of participants (*n* = 8).

Variable	Mean	SD	Lower 95% CI	Upper 95% CI
Age (years)	24.3	3.945	21.47	27.12
Body mass (kg)	75.22	5.919	70.271	80.167
Stature (cm)	171.3	7.2	165.28	177.32
BMI (kg·m^−2^)	25.65	1.698	24.23	27.07
%FM (%)	21.13	4.755	17.16	25.11
Urine specific gravity	1.016	0.008	1.009	1.023

BMI, body mass index; %FM, percentage of body fat; CI, confidence interval.

**Table 2:** Bayesian repeated-measures ANOVA model comparisons for each physiological variable.

Models	P(M)	P(M|Data)	BF_10_
Capillary Glucose (mg/dL)			
Null model (incl. subject)	0.200	0.728	1.000
Condition	0.200	0.235	0.323
Time	0.200	0.028	0.038
Time + Condition	0.200	0.009	0.012
Time + Condition + time ✻ Condition	0.200	<0.001	6.429 × 10^−4^
Peripheral oxygen saturation (SpO_2_)			
Null model (incl. subject)	0.200	<0.001	1000
Condition	0.200	0.017	1.796 × 10^79^
Time	0.200	<0.001	0.011
Time + Condition	0.200	0.727	7.699 × 10^80^
Time + Condition + time ✻ Condition	0.200	0.257	2.719 × 10^80^
Heart Rate (bpm)			
Null model (incl. subject)	0.200	<0.001	1000
Condition	0.200	<0.001	0.400
Time	0.200	0.002	3.933 × 10^65^
Time + Condition	0.200	0.878	1.762 × 10^68^
Time + Condition + time ✻ Condition	0.200	0.121	2.420 × 10^67^
Systolic blood pressure (mmHg)			
Null model (incl. subject)	0.200	<0.001	1000
Condition	0.200	<0.001	2179
Time	0.200	0.025	1.187 × 10^19^
Time + Condition	0.200	0.922	4.380 × 10^20^
Time + Condition + time ✻ Condition	0.200	0.053	2.517 × 10^19^
Diastolic blood pressure (mmHg)			
Null model (incl. subject)	0.200	<0.001	1000
Condition	0.200	<0.001	0.196
Time	0.200	0.815	2.179 × 10^8^
Time + Condition	0.200	0.175	4.689 × 10^7^
Time + Condition + time ✻ Condition	0.200	0.009	2.438 × 10^6^

All models include subject as a random effect, with random slopes specified for all repeated-measures factors. Prior and posterior model probabilities are presented as P(M) and P(M|data), respectively. BF_10_ indicates the Bayes factor for each model.

**Table 3:** Bayesian Inclusion Probabilities and Bayes Factors for Individual Effects.

Effects	P(Incl|Data)	BF Incl
Capillary Glucose (mg/dL)		
Time	0.037	0.026
Condition	0.245	0.216
Time ✻ Condition	<0.001	0.002
Peripheral oxygen saturation (SpO_2_)		
Time	0.983	38.664
Condition	1.000	4.804 × 10^13^
Time ✻ Condition	0.257	1.380
Heart Rate (bpm)		
Time	1.000	2.002 × 10^14^
Condition	0.998	339.696
Time ✻ Condition	0.121	0.548
Systolic blood pressure (mmHg)		
Time	1.000	∞
Condition	0.975	26.012
Time ✻ Condition	0.053	0.224
Diastolic blood pressure (mmHg)		
Time	1.000	1.489 × 10^8^
Condition	0.185	0.151
Time ✻ Condition	0.009	0.037

All models include subject as a random effect. Posterior inclusion probabilities are presented as P(incl|data), and BF incl indicates the Bayesian inclusion factor for each effect.

## Data Availability

Data are available for non-commercial scientific inquiry and/or educational purposes if requested and use does not violate IRB restrictions and/or research agreement terms.

## Abbreviations

The following abbreviations are used in this manuscript:

AOP	Arterial occlusion pressure
BF_10_	Bayes factor (alternative vs. null)
BF incl	Bayesian inclusion Bayes factor
BFR	Blood flow restriction
BMI	Body mass index
CI	Confidence interval
DBP	Diastolic blood pressure
ECG	Electrocardiography
FiO_2_	Fraction of inspired oxygen
HIIT	High-intensity interval training
HR	Heart rate
IPAQ-SF	International Physical Activity Questionnaire—Short Form
ISAK	International Society for the Advancement of Kinanthropometry
NH	Normobaric hypoxia
PAR-Q+	Physical Activity Readiness Questionnaire for Everyone
P(incl|data)	Posterior inclusion probability
P(M)	Prior model probability
P(M|data)	Posterior model probability
SBP	Systolic blood pressure
SpO_2_	Peripheral oxygen saturation
W_max_	Maximal power output

## References

[1] Liang W., Zhu H., Zhao L., Yan X., Lyu Z., Liu C., Huang W. (2025). A time-efficient public health strategy: A systematic review and meta-regression on the comparable and dose-independent effects of sprint interval training vs. moderate-intensity continuous training for metabolic health. Front. Public Health.

[2] Chua M.T., Sim A., Burns S.F. (2022). Acute and Chronic Effects of Blood Flow Restricted High-Intensity Interval Training: A Systematic Review. Sports Med. Open.

[3] Faiss R., Raberin A., Brocherie F., Millet G.P. (2025). Repeated-sprint training in hypoxia: A review with 10 years of perspective. J. Sports Sci..

[4] Patterson S.D., Hughes L., Warmington S., Burr J., Scott B.R., Owens J., Abe T., Nielsen J.L., Libardi C.A., Laurentino G. (2019). Blood Flow Restriction Exercise: Considerations of Methodology, Application, and Safety. Front. Physiol..

[5] Pugh C.F., Paton C.D., Ferguson R.A., Driller M.W., Martyn Beaven C. (2024). Acute physiological responses of blood flow restriction between high-intensity interval repetitions in trained cyclists. Eur. J. Sport Sci..

[6] Conceicao M.S., Junior E.M.M., Telles G.D., Libardi C.A., Castro A., Andrade A.L.L., Brum P.C., Urias U., Kurauti M.A., Junior J.M.C. (2019). Augmented Anabolic Responses after 8-wk Cycling with Blood Flow Restriction. Med. Sci. Sports Exerc..

[7] Pearson S.J., Hussain S.R. (2015). A review on the mechanisms of blood-flow restriction resistance training-induced muscle hypertrophy. Sports Med..

[8] Solsona R., Normand-Gravier T., Borrani F., Bernardi H., Sanchez A.M.J. (2024). DNA methylation changes during a sprint interval exercise performed under normobaric hypoxia or with blood flow restriction: A pilot study in men. Physiol. Rep..

[9] Hughes L., Paton B., Rosenblatt B., Gissane C., Patterson S.D. (2017). Blood flow restriction training in clinical musculoskeletal rehabilitation: A systematic review and meta-analysis. Br. J. Sports Med..

[10] Kasai N., Tanji F., Ishibashi A., Ohnuma H., Takahashi H., Goto K., Suzuki Y. (2021). Augmented muscle glycogen utilization following a single session of sprint training in hypoxia. Eur. J. Appl. Physiol..

[11] Lavigne C., Mons V., Lemineur C., Meste O., Leftheriotis G., Blain G.M. (2025). Physiological mechanisms underlying enhanced performance with blood flow restriction training: Neuromuscular, vascular and metabolic adaptations. J. Physiol..

[12] Millet G.P., Faiss R., Brocherie F., Girard O. (2013). Hypoxic training and team sports: A challenge to traditional methods?. Br. J. Sports Med..

[13] McLean B.D., Buttifant D., Gore C.J., White K., Liess C., Kemp J. (2013). Physiological and performance responses to a preseason altitude-training camp in elite team-sport athletes. Int. J. Sports Physiol. Perform..

[14] Willis S.J., Borrani F., Millet G.P. (2019). Leg- vs arm-cycling repeated sprints with blood flow restriction and systemic hypoxia. Eur. J. Appl. Physiol..

[15] Valenzuela P.L., Sanchez-Martinez G., Torrontegi E., Vazquez-Carrion J., Gonzalez M., Montalvo Z., Millet G.P. (2019). Acute Responses to On-Court Repeated-Sprint Training Performed With Blood Flow Restriction Versus Systemic Hypoxia in Elite Badminton Athletes. Int. J. Sports Physiol. Perform..

[16] Batacan R.B., Duncan M.J., Dalbo V.J., Tucker P.S., Fenning A.S. (2017). Effects of high-intensity interval training on cardiometabolic health: A systematic review and meta-analysis of intervention studies. Br. J. Sports Med..

[17] Hoffmann T.C., Glasziou P.P., Boutron I., Milne R., Perera R., Moher D., Altman D.G., Barbour V., Macdonald H., Johnston M. (2014). Better Reporting of Interventions: Template for Intervention Description and Replication (TIDieR) Checklist and Guide. BMJ.

[18] Richardson A., Twomey R., Watt P., Maxwell N. (2008). Physiological responses to graded acute normobaric hypoxia using an intermittent walking protocol. Wilderness Environ. Med..

[19] World Medical Association (2013). World Medical Association Declaration of Helsinki: Ethical principles for medical research involving human subjects. JAMA.

[20] Pichard C., Kyle U.G., Bracco D., Slosman D.O., Morabia A., Schutz Y. (2000). Reference values of fat-free and fat masses by bioelectrical impedance analysis in 3393 healthy subjects. Nutrition.

[21] Warburton D.E.R., Jamnik V.K., Bredin S.S.D., Gledhill N. (2011). The Physical Activity Readiness Questionnaire for Everyone (PAR-Q+) and Electronic Physical Activity Readiness Medical Examination (ePARmed-X+). Health Fit. J. Can..

[22] Kaspar F., Jelinek H.F., Perkins S., Al-Aubaidy H.A., deJong B., Butkowski E. (2016). Acute-Phase Inflammatory Response to Single-Bout HIIT and Endurance Training: A Comparative Study. Mediat. Inflamm..

[23] Richardson A., Watt P., Maxwell N. (2009). Hydration and the physiological responses to acute normobaric hypoxia. Wilderness Environ. Med..

[24] Buchheit M., Laursen P.B. (2013). High-intensity interval training, solutions to the programming puzzle: Part I: Cardiopulmonary emphasis. Sports Med..

[25] Chua M., Sim A., Burns S.F. (2025). Acute physiological and perceptual responses to three blood flow restricted interval exercise protocols: A randomised controlled trial. Appl. Physiol. Nutr. Metab..

[26] Harwood B., Wright J., Burnet S. (2021). Reliability and validity of the Hypoxico Everest Summit II altitude generator. Proc. Inst. Mech. Eng. Part. P J. Sports Eng. Technol..

[27] Esparza-Ros F., Vaquero-Cristóbal R., Marfell-Jones M. (2019). International Standards for Anthropometric Assessment.

[28] Cannataro R., Cione E., Gallelli L., Marzullo N., Bonilla D.A. (2020). Acute Effects of Supervised Making Weight on Health Markers, Hormones and Body Composition in Muay Thai Fighters. Sports.

[29] Kyle U.G., Bosaeus I., De Lorenzo A.D., Deurenberg P., Elia M., Manuel Gomez J., Lilienthal Heitmann B., Kent-Smith L., Melchior J.C., Pirlich M. (2004). Bioelectrical impedance analysis-part II: Utilization in clinical practice. Clin. Nutr..

[30] American College of Sports Medicine (2013). ACSM’s Health-Related Physical Fitness Assessment Manual.

[31] Ozemek C., Bonikowske A., Christle J., Gallo P. (2025). ACSM’s Guidelines for Exercise Testing and Prescription.

[32] Wagenmakers E.J., Love J., Marsman M., Jamil T., Ly A., Verhagen J., Selker R., Gronau Q.F., Dropmann D., Boutin B. (2018). Bayesian inference for psychology. Part II: Example applications with JASP. Psychon. Bull. Rev..

[33] Team R.C. (2013). R: A Language and Environment for Statistical Computing.

[34] Wickham H. (2016). ggplot2: Elegant Graphics for Data Analysis.

[35] Kay M. (2024). ggdist: Visualizations of Distributions and Uncertainty in the Grammar of Graphics. IEEE Trans. Vis. Comput. Graph..

[36] Wadley G.D., Lee-Young R.S., Canny B.J., Wasuntarawat C., Chen Z.P., Hargreaves M., Kemp B.E., McConell G.K. (2006). Effect of exercise intensity and hypoxia on skeletal muscle AMPK signaling and substrate metabolism in humans. Am. J. Physiol. Endocrinol. Metab..

[37] Richter E.A., Hargreaves M. (2013). Exercise, GLUT4, and skeletal muscle glucose uptake. Physiol. Rev..

[38] Morishima T., Mori A., Sasaki H., Goto K. (2014). Impact of exercise and moderate hypoxia on glycemic regulation and substrate oxidation pattern. PLoS ONE.

[39] Chen H., Liu P., Deng Y., Cai H., Liang P., Jiang X. (2025). The Impact of Blood Flow Restriction Training on Glucose and Lipid Metabolism in Overweight or Obese Adults: A Systematic Review and Meta-Analysis. Life.

[40] De Groote E., Britto F.A., Balan E., Warnier G., Thissen J.P., Nielens H., Sylow L., Deldicque L. (2021). Effect of hypoxic exercise on glucose tolerance in healthy and prediabetic adults. Am. J. Physiol. Endocrinol. Metab..

[41] Kelly L.P., Basset F.A. (2017). Acute Normobaric Hypoxia Increases Post-exercise Lipid Oxidation in Healthy Males. Front. Physiol..

[42] Casey D.P., Joyner M.J. (2012). Compensatory vasodilatation during hypoxic exercise: Mechanisms responsible for matching oxygen supply to demand. J. Physiol..

[43] Spranger M.D., Krishnan A.C., Levy P.D., O’Leary D.S., Smith S.A. (2015). Blood flow restriction training and the exercise pressor reflex: A call for concern. Am. J. Physiol. Heart Circ. Physiol..

[44] Kido K., Suga T., Tanaka D., Honjo T., Homma T., Fujita S., Hamaoka T., Isaka T. (2015). Ischemic preconditioning accelerates muscle deoxygenation dynamics and enhances exercise endurance during the work-to-work test. Physiol. Rep..

